# Receptor Interacting Protein Kinase Pathways Regulate Innate B Cell Developmental Checkpoints But Not Effector Function in Mice

**DOI:** 10.3389/fimmu.2021.758407

**Published:** 2021-12-09

**Authors:** Raksha Parthasarathy, Thomas Hägglöf, Jason T. Hadley, Alexandra McLennan, Aiden Mattke, Elizabeth A. Dudley, Abigail Kumagai, Lily Q. Dong, Elizabeth A. Leadbetter

**Affiliations:** ^1^ Department of Microbiology, Immunology & Molecular Genetics, University of Texas Health at San Antonio, San Antonio, TX, United States; ^2^ Department of Cell Systems and Anatomy, University of Texas Health at San Antonio, San Antonio, TX, United States; ^3^ Department of Engineering, St Mary’s University, San Antonio, TX, United States

**Keywords:** B cell development and differentiation, RIPK1, RIPK3, B-1 cell development, marginal zone B cells, caspase 8

## Abstract

Mutations in the scaffolding domain of Receptor Interacting Protein kinases (RIP) underlie the recently described human autoimmune syndrome, CRIA, characterized by lymphadenopathy, splenomegaly, and autoantibody production. While disease mechanisms for CRIA remain undescribed, RIP kinases work together with caspase-8 to regulate cell death, which is critical for normal differentiation of many cell types. Here, we describe a key role for RIP1 in facilitating innate B cell differentiation and subsequent activation. By comparing RIP1, RIP3, and caspase-8 triple deficient and RIP3, caspase-8 double deficient mice, we identified selective contributions of RIP1 to an accumulation of murine splenic Marginal Zone (MZ) B cells and B1-b cells. We used mixed bone-marrow chimeras to determine that innate B cell commitment required B cell-intrinsic RIP1, RIP3, and caspase-8 sufficiency. RIP1 regulated MZ B cell development rather than differentiation and RIP1 mediates its innate immune effects independent of the RIP1 kinase domain. NP-KLH/alum and NP-Ficoll vaccination of mice doubly deficient in both caspase-8 and RIP3 or deficient in all three proteins (RIP3, caspase-8, and RIP1) revealed uniquely delayed T-dependent and T-independent IgG responses, abnormal splenic germinal center architecture, and reduced extrafollicular plasmablast formation compared to WT mice. Thus, RIP kinases and caspase-8 jointly orchestrate B cell fate and delayed effector function through a B cell-intrinsic mechanism.

## Introduction

Pathogenic missense/nonsense mutations in the Receptor Interactor Protein kinase (RIP) 1 gene have recently been linked to a novel autoinflammatory disease in humans dubbed Cleavage resistant RIP1 Induced Autoinflammatory (CRIA) disease. The disease presents during early childhood and persists through adulthood and is characterized by recurrent fevers lasting 2-7 days along with other symptoms including splenomegaly, lymphadenopathy, abdominal pain, and rashes as well as increased levels of proinflammatory cytokines in the serum (1). At the same time, another study has linked biallelic mutations in human RIP1 to inflammatory bowel disease and combined immunodeficiency through altered inflammasome and NFκB activity (2, 3). Several mouse models implicate a role for RIP1 and RIP3 in human diseases like epidermal hyperplasia, liver injury, and atherosclerosis (4); however, a specific cellular mechanism of action of RIP1 in these disease models remains elusive.

RIP1 is an adapter molecule which partners with RIP3 and/or caspase-8 to act downstream of the TNF family of receptors and toll-like receptors. RIP1 can play contrasting roles directing cell death or mediating pro-survival signals depending on context. Together with caspase-8, RIP1 conveys signals for apoptotic cell death while RIP1 and RIP3 interactions mediate signaling for necroptosis. On the other hand, ubiquitinated RIP1 can mediate pro-survival signals through NFκB gene expression (5). RIP1 has a kinase domain (KD), death domain (DD), and a RIP homotypic interaction motif (RHIM). RIP3, a kinase in the same family, lacks the DD and is essential for necroptotic cell death. The scaffold domain of RIP1 can prevent kinase independent activation of caspase-8 and RIP3, adding another layer of signal regulation (6).

The role of RIP kinases in innate immunity and in the context of certain viral infections has been well documented. During an active infection, vaccinia virus activates RHIM-dependent necroptosis in host cells leading to anti-viral inflammation (7). RIP1 plays a further role in immune effector function by engaging NFκB to drive cytokine production in BMDMs (8) and partnering with RIP3 for the production of pro-inflammatory cytokines in macrophages *via* Erk1/2 (9). So, RIP1 mediates certain leukocyte effector functions, but the role of RIP1 in leukocyte development remains unexplored. On the other hand, RIP3 has been demonstrated to be dispensable for macrophage, natural killer cell, and lymphocyte development (10).

Caspase-8, a proapoptotic protease, also plays a vital role in immune defense by enabling infected cells to die an inflammatory death which subsequently recruits protective lymphocytes (11). In fact, caspase-mediated cell death is so important to viral immune defense, it is specifically targeted by large DNA viruses as a means to evade host immune defenses and facilitate viral persistence (12). *In vivo* conditional knockout of caspase-8 under the Mx1 promoter affects T cell development in the thymus (13). However, caspase-8 deletion under the Lck promoter does not affect T cell development in the thymus but reduces peripheral T cell numbers (14) indicating a possible role for caspase-8 in early T cell maintenance. Similarly, caspase-8 deficient B cells developed normally, but failed to proliferate upon LPS or dsRNA stimulation and had impaired antibody production (15). An early study was initially interpreted to show that caspase-8 mediates signals from the antigen receptor to NFκB in T, B, and NK cells from both humans and mice (16). However, more recent studies have implicated RIP3 and MLKL-mediated necroptosis as responsible for the proliferation defects observed in Caspase-8 knock-out mice (17). In short, caspase-8 plays a key role in antigen receptor signaling and lymphocyte effector function, which may be different from its role in mediating programmed cell death (16).

Dissecting the role of RIP1 in immune cells has been complicated by the fact that RIP1 deficient mice do not survive to adulthood. However, mice lacking RIP1, RIP3, and caspase-8 (TKO; *Casp8^-/-^, Ripk3^-/-^, Ripk1^-/-^*) will survive to adulthood, so it is possible to study the contribution of RIP1 against the backdrop of caspase-8 and RIP3 deficiency. Comparing TKO mice to double knock out mice lacking RIP3 and caspase-8 (DKO; *Ripk3^-/-^, Casp8^-/-^*) we can determine RIP1’s relative role in lymphocyte differentiation and maintenance. In the TKO mice, abnormal B220^+^ CD3^+^ T cells develop and accumulate with age (18). This is reminiscent of a similar autoimmune T cell population in Fas/FasL deficient mouse models (19). TKO mice have total T cell numbers similar to WT mice, but their CD4:CD8 T cell ratios are skewed and specialized T cell subsets have not been investigated in great detail (17, 18, 20). Early studies did not identify any differences in total B cells between TKO and wild-type control mice (18).

TNF-receptor superfamily ligands mediate important aspects of B cell development, maintenance, and homeostasis. For example, FAS signaling induces extrinsic cell death during B cell differentiation to eliminate self-reactive cells and prevent development of autoimmunity (19). On the other hand, BAFF-R signals through non-canonical NFκB are critical for maintaining B cell survival (21, 22). Accordingly, targeted disruption of NFκB signaling can lead to defects in B cell development in the bone marrow and differentiation in the periphery, impairing maturation of transitional immature B cells (23). Ample evidence places RIP1 downstream of TNF receptors (24) and recent studies have characterized a role for RIP1 in mediating signals downstream of T cell receptors (TcRs). First, despite normal numbers at 4 weeks, few mature T cells were sustained when immuno-deficient mice are reconstituted with RIP1-deficient fetal liver cells (25, 26), suggesting RIP1 signals are critical for T cell development. More specifically, RIP1 is induced in thymocytes following positive selection, a step mediated by TcR recognition of MHC, but survival of those thymocytes requires that cell-death activity of RIP1 can be repressed by IKK (27). The IKK signal is secondary to TNFR engagement, a capability restricted to single positive thymocytes. In this way, RIP1 mediates survival of thymic precursors which have received engagement of both TcR and TNFR. Mice with T cell-restricted deletion of RIP1 have severe T cell development defects and secondary peripheral lymphopenia (28). Since the same T cell developmental defects were not observed in mice with a selective mutation preventing RIP1 kinase activity, these functions are likely mediated by RIP1’s scaffolding function (28). T cells from these mice proliferated poorly in response to TcR engagement and have defective TcR-induced p65 NFκB phosphorylation (29) consistent with a defect in TcR signaling. This places RIP1 as a key rheostat downstream of TcR signaling, mediating T cell selection, survival, and death in the thymus. As described, RIP1 has been implicated in mediating T cell development and TcR signaling, but initial studies ruled out a parallel role of RIP1 in B cells. However, more recent studies found RIP1 can be tied to changes in B cell development. RIP1 expression starts at a low level in bone marrow pre/pro B cells but increases in immature B cells and reaches the highest level in mature peripheral B cells (29). In an interesting parallel to T cell development, wild-type mice reconstituted with RIP1-deficient BM showed reduced IgM^+^ IgD^lo^ (MZ) B cell frequencies within 7 weeks, with those frequencies dropping even further by the 12th week until IgM^+^ IgD^+^ B cells were almost undetectable (29). These same authors found fetal liver-derived RIP1-deficient B cells proliferate and upregulate activation markers normally in response to *in vitro* stimulation with anti-IgM, anti-CD40, and TLR9 antigen, but had reduced responses to TLR2, 3, and 4 engagement. This outcome fits with the connection between RIP1 and TNFR mediated survival of leukocytes during development.

Given the important regulation mediated by TNF-superfamily signals through the RIP kinase pathway, we hypothesized that a deficiency in RIP1/RIP3/caspase-8 would impact B cell survival and homeostasis and examined the number and function of various B cell subsets independent of the apoptosis pathway. Our preliminary data suggests that the absence of RIP1 leads to selective changes in innate B cell subsets which is consistent with RIP1 mediating antigen-selection and signal strength downstream of the B cell receptor (BcR) or BAFF, similar to the role it plays in T cell development. Indeed, innate B cells (MZ B cells and B-1b cells) are altered in frequency and number and show modest delays in antigen responsiveness in both TKO and DKO mice. These alterations suggest that RIP1 signaling downstream of the BcR may translate the strength of BcR signals to the NFκB pathway to mediate B cell selection, maintenance, and responsiveness.

## Materials and Methods

### Mice

RIP3-/-, DKO, TKO and RIP1kd mice were a gift from Dr. William Kaiser (UT Health San Antonio). B6.SJL-ptprc^a^ Pepc^b^/BoyJ (Pepboy) and B6.129S2-Ighm^tm1Cgn^/J (μMT) were purchased from the Jackson Laboratory (Bar Harbor, ME). C57BL/6 wild-type mice were housed and bred under specific pathogen-free conditions and all mice were maintained at the Laboratory Animal Resources animal facility of UT Health San Antonio. All experiments were performed under the UT Health San Antonio LAR and IACUC approved protocols. Age- and sex-matched female or male mice were used in all experiments, primarily aged 6-14 weeks, with a few experiments using mice up to 28 weeks, as noted in the legends. Littermates were randomly assigned to experimental treatment groups. Group sizes were determined based on previous experience, investigators were not blinded.

### Immunofluorescence

6μm-8μm sections of optimal cutting temperature (OCT compound, Fisher Healthcare)-embedded murine spleen and kidneys were cut using a cryostat NX (Thermo Scientific) and immediately fixed in acetone before blocking with 5% FBS/PBS. Primary conjugated antibodies (TCRβ-PE, IgD-FITC, CD1d-AF647, CD35-APC, NP-PE, GL7-FITC, CD138-APC, IgG1-FITC; BD Biosciences and BioLegend) were used to label tissue sections, followed by 5% FBS/PBS wash. Coverslips were mounted using Prolong™ Glass Antifade Mount with NucBlue (Invitrogen) and sections were imaged using a Zeiss fluorescent microscope. Analysis included 3-5 images per section, multiple sections per spleen, multiple animals per organ, as noted in figure legends.

### ELISA

ELISA was used to detect antigen-specific IgM, IgG1, IgG2b, and IgG3 titers by capturing serum antibodies using NIP_11_-OVA (Jackson ImmunoResearch, Inc) coated plates (NP-specific) or methylated BSA and calf thymus DNA (Sigma-Aldrich) coated plates (DNA-specific), as described previously (30, 31). Bound antibody was detected with anti-IgM-HRP, anti-IgG1-HRP, anti-IgG2b-HRP or anti-IgG3-HRP (Southern Biotech), TMB substrate (Biolegend), and read on a Molecular Devices microplate reader at 450nm. Titer was determined to be lowest serum dilution achieving signal greater than 2x background. BAFF in sera was measured by a sandwich ELISA using BAFF- specific clones 5A8 (capture) and 1C9-biotin (detection) (Axxora) as performed previously (31). Recombinant mouse BAFF (R&D Systems) served as standard.

### Flow Cytometry

3x10^6^ murine splenocytes or peritoneal washout cells per sample were treated with ACK lysis buffer (Lonza), blocked with anti-mouse CD16/32 (clone 93) to reduce non-specific labeling, and stained with Live/Dead Fixable Zombie viability dye (BD Biosciences) to exclude dead cells. Further surface staining was performed using the following antibodies (BD Pharmingen, Biolegend, R&D Systems, or eBiosciences): CD21/35 (7G6), CD23 (B3B4), CD95 (Jo2), CD138 (281-2), GL7, Ly6G (1A8), CD11b (M1/70), B220 (RA3-6B2), IgM (RMM-1), IgD (11-26c.2a), BAFF (1C9), BAFF (121808), CD1d (1B1), CD18 (H155-78), CD62-L (MEL-14), CD8α (53-6.7), TCRβ(H57-597), FasL (MFL-3), CD45.1 (A20), CD45.2 (104), CD5 (53-7.3), CD69 (H1.2F3), CD19 (6D5). Doublet exclusion and live-cell discrimination was performed on all cell populations prior to sub-gating. Samples were acquired on a BD FACS Celesta (Becton Dickinson) at the UT Health San Antonio flow cytometry core and analyzed using FlowJo software (10.5.3).

### Bone Marrow Chimeras

To create mice with cell death pathway kinase deficiency primarily restricted to the B cell subset, mixed BM chimeras were created by mixing 20% C57BL/6 wild-type, RIP3-/-, DKO, TKO or RIP1^kd^ bone marrow with 80% μMT (B6.129S2-Ighm^tm1Cgn^/J) bone marrow and 5 × 10^6^ cells were transferring to groups of 6, 8-10 week old female B6.SJL-ptprc^a^ Pepc^b^/BoyJ recipient mice. Prior to transfer, recipients were irradiated twice, for a total of 900 rads, 2 hours apart, and rested for a few hours before reconstitution. Enroflox 100 was added to water of the recipient mice for 2 weeks post irradiation, and mice were reconstituted for 8-10 weeks prior to evaluation. B6.SJL-ptprc^a^ Pepc^b^/BoyJ and unirradiated C57BL/6 wild-type mice were used as flow cytometry controls.

### Infection

All infection experiments were carried out in a BSL-2 facility with IACUC and Biosafety approval. *Streptococcus pneumoniae* URF 918 was grown on blood agar plates overnight, then collected and regrown to log phase in Todd Hewitt broth culture supplemented with yeast extract. Mice were systemically infected with 6-10 x 10^5^ CFU/mL of *Streptococcus pneumoniae* at mid log phase i.v. and then observed for 7-12 days. Infectious dose was confirmed by culturing 3 log serial dilutions of inoculum on blood agar plates for 24 hours.

### Immunoblotting

B cells were negatively isolated from naive C57BL/6 wild-type murine spleen using the EasySep™ Mouse B cell isolation kit (Cat# 19854, Stemcell Technologies) according to manufacturer’s instructions. 2x10^6^ B cells were plated per well and *in vitro* cultured with 15ug/mL anti-IgM F(ab’)_2_ (Cat# 115-006-020, Jackson ImmunoResearch), 10ug/mL of LPS, for 5-15 minutes. Cells were pretreated with 39.5uM RIPK inhibitor, Necrostatin-1 (Cat# N9037, Sigma-Aldrich) for 60 minutes as indicated. Following culture, cells were lysed using 2x SDS sample buffer and heated to 95°C for 5 minutes. Proteins were separated by sodium dodecyl sulphate polyacrylamide gel electrophoresis (SDS-PAGE) using a 12.5% resolving gel run at 100V for ~2.5 hours. Gels were transferred to nitrocellulose membranes, 0.2μm (BioRad; #10484059) at 0.4A for 2hrs and blocked in 1% non-fat dry milk for 1 hour at RT. Proteins were detected by immunoblotting cell lysates with antibodies specific for p-p65 and p65 (#8242T, #3033T; Cell Signaling and Technology) or β-actin (# MAB8929SP; R&D Systems) and imaged using the syngene G:box image system. The relative increase in protein phosphorylation (expressed as percentage of basal phosphorylation; Arbitrarily set as 1.0) was quantified by ImageJ software. All results are presented as mean with SD and p-values were calculated using one-way ANOVA.

### Statistical Analysis

Statistical significance was assessed by GraphPad PRISM 7 using a two-tailed Student’s *t*-test, paired *t*-test, one-way ANOVA, or Kaplan -Meier survival analysis as indicated in figure legends. *P ≤*0.05 is considered statistically significant. Variance is similar between all groups compared, and data points were excluded from consideration if identified by the ROUT method using Graphpad PRISM software. Box heights and circle, triangle, box centers indicate group means and/or individual values. Error bars indicate s.e.m.

## Results

### RIP1 and Caspase-8 Control Peripheral Innate B Cell Homeostasis

*Ripk1* ^–/–^ mice die shortly after birth (32) and mice with *Casp8*-deficiency do not survive embryogenesis. However, *Casp8* ^–/–^ mice can be rescued by *Ripk3* depletion to produce viable mice with a normal mendelian frequency of offspring capable of living well into adulthood (17, 20). So, in order to study the role of RIP kinases and caspase-8-mediated signaling pathways in innate B cell development *in vivo*, we used mice lacking *Ripk3* (RIP3-/-), double knockout mice lacking both *Ripk3* and *Casp8* (DKO), triple knock out mice lacking *Ripk3*, *Ripk1* and *Casp8* (TKO), or *Ripk1^K45A/K45A^* mice (RIP1^kd^) which have a point mutation (K45A) knocked-in which renders the RIP1 kinase domain inactive but still retains its scaffolding function. In wild-type mice, caspase-8 negatively regulates RIP1, so RIP1 will be unrestricted and possibly overactive in DKO mice lacking both RIP3 and caspase-8 (33). Conversely, TKO mice lack both RIP3 and caspase-8 in addition to lacking RIP1, so instead of enhanced RIP1 activity, TKO mice exhibit reduced/absent RIP1 activity.

**Figure 1:**
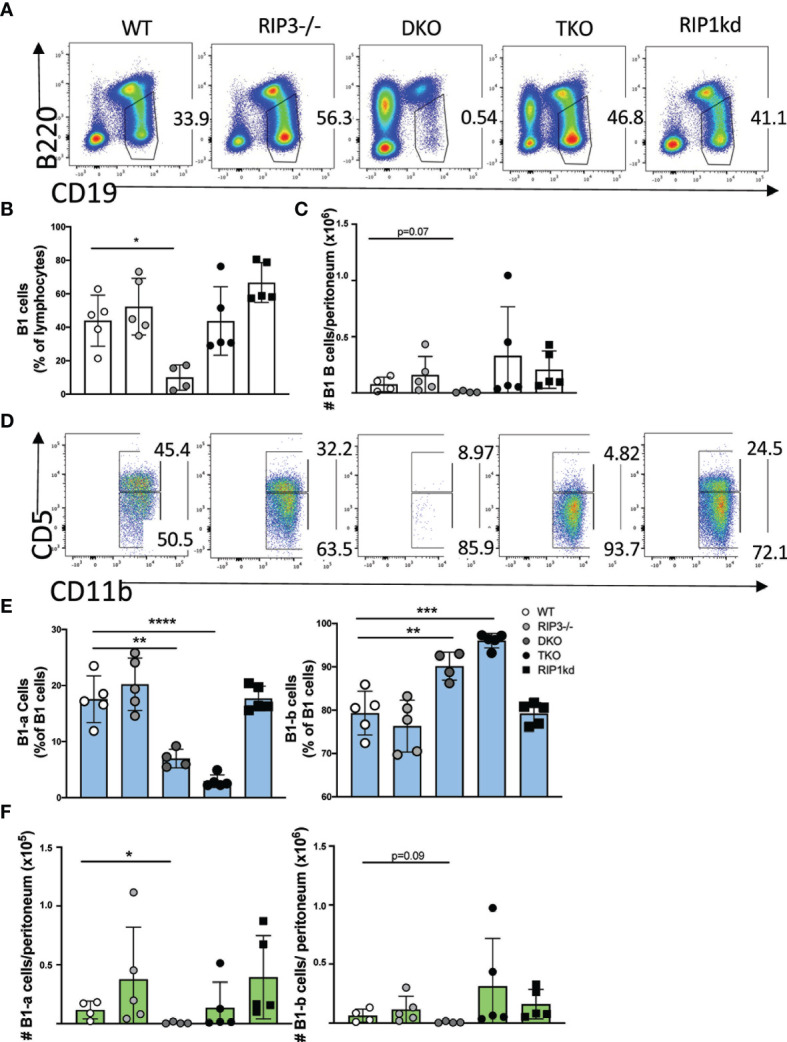
RIP1 and caspase-8 are required for innate B-1 B cell homeostasis. Flow cytometry of C57BL/6 WT, RIP3-/-, DKO, TKO and RIP1^kd^ murine peritoneal washout cells identifies CD19^+^B220^lo^ B1 B cells as percent of live lymphocytes **(A)**. Scatterplot quantifies frequency **(B)** and number **(C)** of peritoneal total CD19^+^ B220^lo^ B-1 cells. From the CD19^+^B220^lo^ population, CD11b^+^IgM^+^ B-1 cells (gate not shown) were subgated to identify B1-a (CD5^+^) or B1-b (CD5^-^) subsets **(D)**. Scatterplots quantify frequency **(E)** and number **(F)** of peritoneal B1-a (left) and B1-b (right) cells. Data is representative **(A, D)** or a pool **(B, C, E, F)** of two independent experiments with 2-3 mice per group, 8-14 weeks old. Each symbol indicates an individual mouse. *p ≤ 0.05, **p ≤ 0.01, ***p ≤ 0.001, ****p ≤ 0.0001 (One-way ANOVA).

We first used multicolor flow cytometry to determine B cell subset distribution in mice lacking various components of RIP kinase and caspase-8 pathways. Importantly, for these examinations we considered the frequency of lymphocyte populations by excluding from consideration the autoimmune CD3^+^B220^+^ population which develops with age in DKO and TKO mice (17, 20). After excluding the CD3^+^B220^+^ T cell autoimmune population, the number of total conventional B cells (CD19^+^B220^+^ or CD19+ in one case) in the spleens were comparable between WT, DKO, and TKO mouse strains (**Supplementary Figures 1A, B**). With that consideration, we next looked at an innate B cell population and found that that B1 B cells, defined for flow cytometry as CD19^+^ B220^lo^ (**Figure 1A**), made up a lower frequency (and trended towards a significantly lower number) of total lymphocytes in peritoneal washouts of DKO mice than all other strains of mice investigated (**Figures 1B, C**). Interestingly, while DKO mice were lacking almost all B1 cells, in TKO mice, B1 B cells represent a similar frequency of the total B cell population and were present at similar numbers to those found in WT, RIP3-deficient mice, or RIP1kd control mice (**Figures 1B, C**). However, within the B1 B cell population, TKO mice possess an altered ratio of B1-a to B1-b cells, containing significantly lower frequencies of B1-a cells (CD19^+^IgM^+^CD11b^+^CD5^+^) and higher frequencies of B1-b cells (CD19^+^IgM^+^CD11b^+^CD5^–^) when compared to RIP3-deficient mice, RIP1^kd^ mice, and wild-type control mice (**Figures 1D, E**). While DKO mice have very few peritoneal B-1 cells, those that are able to develop have a disrupted ratio similar to the TKO, with lower frequencies and numbers of B1-a cells and higher frequencies of B1-b cells (**Figures 1D–F**).

**Figure 2:**
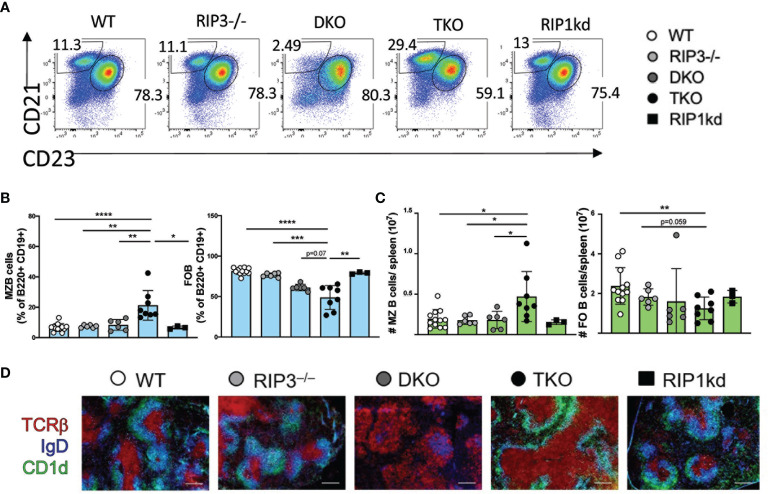
RIP1 and caspase-8 are required for innate MZ B cell homeostasis. Flow cytometry of C57BL/6 WT, RIP3-/-, DKO, TKO and RIP1^kd^ splenocytes identifies percent of B220^+^, CD19^+^ B cells which are CD21^hi^ CD23^lo^ MZ B cells and CD21^lo^ CD23^hi^ FO B cells as indicated on plots **(A)** and quantified in **(B)**. Number of MZ B cells and FO B cells per spleen are quantified in **(C)**. Immunofluorescent labeling of 8μm thick spleen sections from C57BL/6 WT, RIP3-/-, DKO, TKO and RIP1^kd^ mice identifies the T cell zone (anti-TCRb), B cell follicle (anti-IgD), and the marginal zone (anti-CD1d) **(D)**. Data is representative **(A, D)** or a pool **(B, C)** of three independent experiments with 2-4 mice per group, 8-14 weeks old. Each symbol indicates an individual mouse. *p ≤ 0.05, **p ≤ 0.01, ***p ≤ 0.001, ****p ≤ 0.0001 (One-way ANOVA). Scale bar=200μm.

We next considered another innate B cell population, marginal zone (MZ) B cells, in the spleen. Again, by excluding the autoimmune CD3+B220+ T cell population, we found that within the CD19^+^/CD19^+^B220^+^ B cell population, TKO mice exhibited a significantly expanded frequency and number of CD21^hi^ CD23^lo^ splenic marginal zone B (MZ) B cells vs a contracted frequency and number of follicular (FO) B cells when compared to wild-type controls and most other groups (**Figures 2A–C**). When MZ B cells are activated during inflammation, they are able to shed CD21 expressed on the surface and thus adopt a phenotype more similar to FO B cells (34). In order to confirm that changes in MZ B cell frequencies were not simply due to downregulation/shedding of surface CD21, we also characterized frequencies of splenic MZ B cells and FO B cells using CD1d and CD23. We found similar increases in MZ B cells from TKO mice compared to wild-type controls when identifying MZ B cells using CD1d^hi^ CD23^lo^ markers (**Supplementary Figures 1B, C**), suggesting that MZ B cell development was impaired rather than the population being phenotypically altered due to chronic activation in the TKO mice. To determine if the altered MZ B cell frequencies were a consequence of innate B cell developmental defects, we next assessed frequencies of MZ B cell precursors: Transitional stage 1 B cells (T1 cells; CD23^-^, CD21^lo^, IgM^hi^) and Transitional stage 2 cells (T2 cells; CD23^+^, CD21^hi^, IgM^hi^) (**Supplementary Figures 1D, E**). DKO mice had a significantly increased frequency of T1 B cells but no change in frequency of T2 cells (**Supplementary Figure 1E**). In fact, the T2 B cell frequency remained constant across all genotypes (**Supplementary Figure 1E**). NOTCH-2 is a key protein indispensable for MZ B cell development (35), so we also measured NOTCH-2 expression on splenic T2 cells in RIP deficient mice. TKO mice had significantly higher frequency of NOTCH-2+ T2 cells when compared to wild-type and DKO mice (**Supplementary Figure 1F**) suggesting RIP1 plays a role in B cell development. To confirm that the changes in frequency of MZ B cells and FO B cells were independent of impaired proliferation or cell death, we assessed the proliferative characteristics of T1, T2, MZ B cells and FO B cells. T1 cells showed modestly decreased proliferation in both DKO and TKO mice, but only exhibited increased cell death in TKO mice (**Supplementary Figure 1G**). There were no changes in the proliferative marker Ki67 nor in the frequency of dead T2, MZ, or FO B cell populations in DKO and TKO mice compared to RIP3-/- mice, RIP1^kd^ mice and wild-type controls (**Supplementary Figures 1H, I**). This is consistent with RIP1 affecting proliferation and/or survival of the early T1 population, but the changes observed in MZ B cells and FO B cells in DKO and TKO mice being independent of proliferative or survival effects.

Next, we used immunofluorescence to assess whether changes in splenic B cell populations are reflected by changes in splenic architecture. It is well documented that both TKO and DKO mice have an expanded population of atypical CD3^+^B220^+^ T cells that are likely to be autoreactive (18, 20). These atypical T cells are highly prevalent in the spleen, and their presence coincides with a highly disrupted splenic architecture (**Figure 2D**). Consistent with our flow cytometry data, immunofluorescent labeling of spleen tissue revealed that TKO mice also had an enlarged MZ B cell zone while DKO mice had almost undetectable MZ B cell population, compared to wild-type mice (**Figure 2D**). Thus, RIP1- and caspase-8-deficiencies drive an imbalance in frequencies and numbers of innate B cells with TKO mice developing increased MZ B cells and B1 B cells skewed towards the B1-b subset, as compared to wild-type mice. DKO mice lack B1 cells entirely and display a lower frequency and number of MZ B cells than wild-type mice. These differences in B cell populations are consistent with RIP kinases and caspase-8 mediating signals which dictate B cell population identity.

### *Ripk1* and *Casp8* Deficiency Allows Increased BAFF Expression by Macrophages

**Figure 3:**
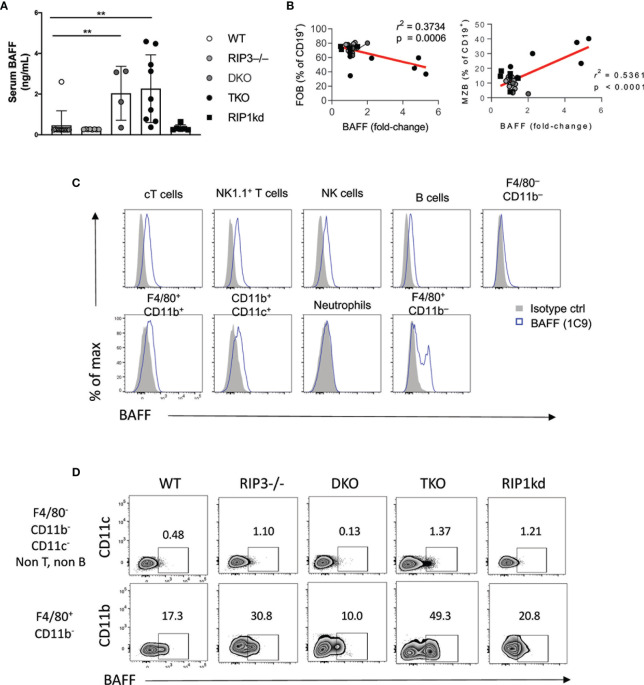
RIP1 and caspase-8 restrain BAFF production by myeloid cells. ELISA detects BAFF in serum from C57BL/6 WT, RIP3-/-, DKO, TKO and RIP1^kd^ mice **(A)**. Serum BAFF levels correlate with FO B cell (left) and MZ B cell (right) frequencies **(B)**. Flow cytometry reveals intracellular BAFF in indicated lymphocyte subsets labeled with anti-BAFF (1C9) or isotype control **(C)**. Flow cytometry further identified intracellular BAFF in F4/80^+^ and F4/80^-^ cells from C57BL/6 WT, RIP3-/-, DKO, TKO and RIP1^kd^ mice **(D)**. Data is representative **(C, D)** or a pool **(A, B)** of two independent experiments with 2-4 mice per group, aged 8-28 weeks. Each symbol indicates an individual mouse. **p ≤ 0.01 (One-way ANOVA, Unpaired two-tailed test and simple linear regression).

B cell activating factor (BAFF), a TNF ligand superfamily cytokine, interacts with BAFF-receptor (BAFF-R), BCMA, and TACI to provide essential maintenance signals for B cells (36). BAFF-transgenic mice have an increased frequency of MZ B cells (37, 38). Given the importance of BAFF in innate B cell maintenance, we next used an ELISA to measure the amount of BAFF in the serum of RIP kinase- and caspase-8 deficient mice. DKO and TKO mice had significantly higher serum BAFF levels than wild-type, RIP3-/-, and RIP1^kd^ mice (**Figure 3A**). Across all strains, FO B cell frequencies negatively correlated and MZ B cell frequencies positively correlated with serum BAFF levels (**Figure 3B**). These findings are consistent with the critical role for serum BAFF in maintaining MZ B cells (37, 39, 40), and the increased serum BAFF in TKO mice may help to maintain their higher frequency and number of MZ B cells.

To identify possible sources of BAFF in these mice, we used flow cytometry to examine expression of BAFF by different splenic leukocyte subsets. BAFF is expressed by various cell types including but not limited to conventional T cells (cT), NKT cells, macrophages, and even B cells (**Figure 3C**) (41, 42). Of the cells tested, macrophages were the population with the highest frequency of BAFF^+^ cells across all murine strains. Upon closer examination, flow cytometry showed a higher frequency of BAFF^+^ F4/80^+^ CD11b^–^ macrophages and F4/80^-^ CD11b^-^ CD11c^-^ macrophages in TKO mice compared to wild-type, RIP3-/- and RIP1^kd^ control mice (**Figure 3D**).

High concentrations of serum BAFF is a characteristic feature of autoimmune diseases and is consistent with the autoimmune symptoms previously reported in TKO mice (18, 43). To assess autoimmune features associated with increased amounts of BAFF in serum, we next measured deposition of IgG1 immune complexes in kidneys and assessed autoreactive antibody titers in serum. Immunofluorescent staining detected significant IgG1 deposition in the kidneys of TKO and DKO mice as compared to wild-type mice (**Supplementary Figure 2A**). Furthermore, anti-DNA antibody ELISAs detected significantly higher titers of autoantibodies in the serum of TKO and DKO mice as compared to wild-type controls (**Supplementary Figure 2B**). RIPK1kd mice had some variability across their response, but it was not significantly different than the intact controls (**Supplementary Figure 2B**). Thus, TKO and DKO mice show the highest serum amounts of BAFF and the TKO mice had a concomitant increase in the frequency of BAFF-expressing F4/80^+^ macrophages compared to wild-type mice. These findings are consistent with macrophages producing BAFF, which supports innate B cells and contributes to autoimmunity.

### Mixed BM Chimeras Reveal That Limiting *Ripk1*- and *Casp8*- Deficiency Primarily to B Cells Restores MZ B Cell Number and Splenic Architecture

**Figure 4:**
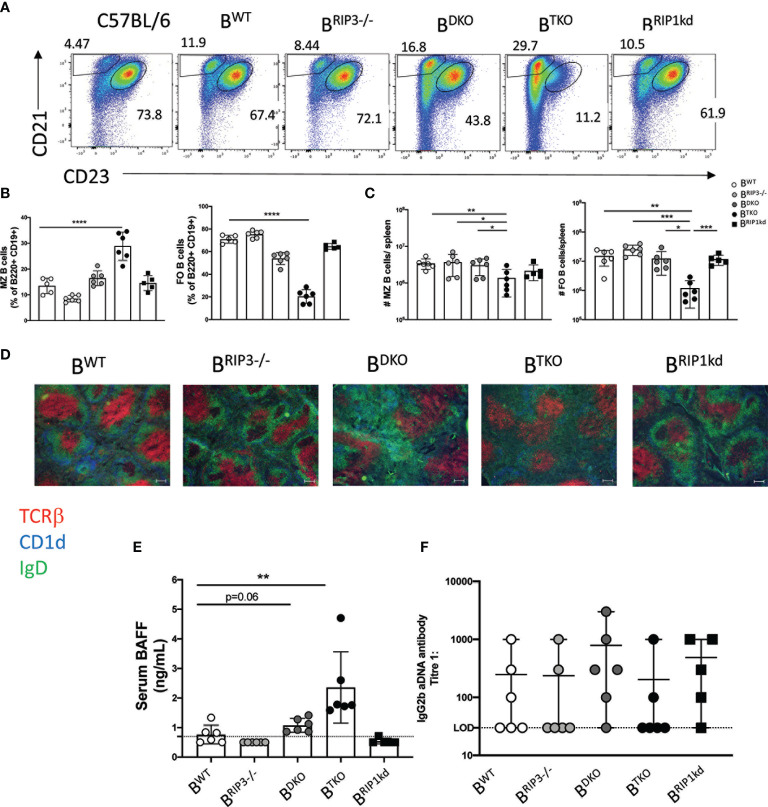
Intrinsic RIP1 and caspase-8 is required for innate MZ B cell homeostasis. Flow cytometry reveals frequency of CD45.2^+^B220^+^CD19^+^ B cells that are MZ B cells (CD21^hi^ CD23^lo^) vs FO B cells (CD21^lo^CD23^hi^) in spleens from B^WT^, B^RIP3-/-^, B^DKO^, B^TKO^ and B^RIP1kd^ mixed bone marrow chimeras 10 weeks post reconstitution **(A)**, quantified in **(B)**. Donor MZ B cells and FO B cells numbers per spleen are shown in **(C)**. Immunofluorescent images of 6μm thick spleen sections from mice in **(A)** labeled to identify T cell zone (anti-TCRβ; red), B cell zone (anti-IgD; green), and Marginal zone (anti-CD1d; blue) **(D)**. ELISA detected serum BAFF from B^WT^, B^RIP3-/-^, B^DKO^, B^TKO^ and B^RIP1kd^ mice 10 weeks post reconstitution **(E)**. ELISA detected IgG2b serum autoantibody titers in B^WT^, B^RIP3-/-^, B^DKO^, B^TKO^ and B^RIP1kd^ mice **(F)**. Data is representative **(A, D)** or a pool **(B, C, E, F)** of two- three independent experiments, 2-4 mice per group. Each symbol indicates an individual mouse. *p ≤ 0.05, **p ≤ 0.01, ***p ≤ 0.001, ****p ≤ 0.0001 (One-way ANOVA and Non-parametric Unpaired two-tailed t-test). Scale bar=100μm.

Since systemic absence of RIP1 and caspase-8 leads to increased MZ B cell frequencies, we next asked whether B cell-intrinsic expression of RIP1 and caspase-8 was required to support MZ B cell differentiation, or if stromal cell expression was sufficient. To this end, we generated mixed bone marrow chimeras (BMC) which restrict the absence of RIP3 and caspase-8 (DKO) or RIP3, caspase-8, and RIP1 (TKO) primarily to CD45.2 congenically marked B cells (**Supplementary Figure 3A**). We engrafted CD45.1 congenic wild-type host mice with a 5:1 ratio of B cell-deficient μMT BM, and either wild-type RIP3-/-, DKO, TKO or RIP1^kd^ BM from CD45.2 congenic mice. After 10 weeks of reconstitution, we characterized B cell splenic subsets by flow cytometry. Interestingly, we found that B cells in general were slow to repopulate the BM chimeras reconstituted with TKO BM (B^TKO^ mice). B cells represented a lower frequency of splenic lymphocytes and were present in lower numbers in B^TKO^ mice than in BM chimeras repopulated with BM from all the other strains (**Supplementary Figures 3B, C**). The B220^+^CD3^+^ T cell population also expanded significantly in number and frequency in the spleens of mice with BM primarily from DKO mice (B^DKO^) and B^TKO^ mice, but to a much lower extent in the B^TKO^ mice (**Supplementary Figures 3D, E**). These autoreactive T cells were excluded from our B cell analysis. We first used two different antibody combinations to characterize MZ B cell frequencies (**Figure 4A** and **Supplementary Figure 3F**). Strikingly, neither the frequency nor number of MZ B cells in the B cell compartment of BMC mice with DKO B cells (B^DKO^) significantly differed from B^WT^ controls (**Figures 4A–C** and **Supplementary Figures 3G–J**). On the other hand, we found that in B^TKO^ mice, splenic MZ B cells represented a higher frequency, and FO B cells a commensurate lower frequency, of the CD19^+^B220^+^ B cell population than MZ and FO B cells in control mice reconstituted with wild-type B cells (B^WT^) (**Figures 4A, B** and **Supplementary Figures 3G, H**). Because CD19^+^B220^+^ B cells in B^TKO^ mice were delayed or reduced following BM reconstitution (**Supplementary Figures 3B, C**), the numbers of both MZ and FO B cells are reduced in B^TKO^ BMC as compared to B^WT^ and other controls (**Figure 4C** and **Supplementary Figures 3I, J**). However, MZ B cell numbers are reduced to a lesser extent than the FO B cells in B^TKO^ mice, consistent with the increase in relative percentage of MZB cells in B^TKO^ mice (**Figures 4B, C** and **Supplementary Figures 3G–J**). To determine if the increase in MZ B cell frequency in the B^TKO^ BMC mice was a consequence of developmental and/or maintenance defect in precursor populations, we measured T1 and T2 B cell frequencies by flow cytometry. Interestingly, the B cell compartment in the B^TKO^ mice exhibited a trend towards a reduction in the number of T1 cells compared to B^WT^ and other controls but the differences were not significant (**Supplementary Figures 3K–N**). The absence of differences in T1 and T2 cell frequencies in the BM chimera mice may be attributed to the duration of bone marrow reconstitution.

We next used immunofluorescent imaging to determine whether B cell-intrinsic RIP1 and caspase-8-deficiency affects lymphocyte architecture in the spleen. Similar to mice with systemic deficiencies, B^TKO^ BMC showed an accumulation of MZ B cells, consistent with flow cytometry data (**Figure 4D**), although the general splenic architecture of all BM chimera groups was more similar to wild-type controls than kinase deficient mice. Surprisingly, the autoreactive T cell overgrowth observed in DKO and TKO mice was also replicated in B^TKO^ and B^DKO^ BMC when compared to B^WT^ BMC controls (**Supplementary Figure 3C**). To assess the autoimmune phenotype of the BMC mice, we measured BAFF and autoantibodies in the serum of BMC. B^TKO^ BMC had significantly higher and B^DKO^ BMC had trending higher concentrations of serum BAFF when compared to the B^WT^ BMC, which replicates the phenotype observed in the unmanipulated DKO and TKO mice (**Figure 3A** and **Figure 4E**). Autoantibodies in serum did not significantly differ between the BMC groups (**Figure 4F**), but this may reflect the younger age of the immune system in BMC as compared to intact adult mice. Thus, B cell-intrinsic RIP1 and caspase-8 are required to maintain homeostatic MZ B cell frequency and numbers, but do not dictate early developmental intra-splenic localization and autoantibody production.

### *Casp8* Is Required for T-Dependent B Cell Expansion, Germinal Center Differentiation, and Timely Antibody Responses

MZ B cells shuttle antigens to follicular dendritic cells as part of an efficient adaptive immune response (44). Given the imbalance in splenic MZ B cell and FO B cell frequencies observed in DKO and TKO mice, we next investigated whether these changes would impact functional adaptive immune responses. First, we measured adaptive B cell responses in the RIP3-/-, DKO, TKO, and RIP1^kd^ mice by immunizing them with the T-dependent (TD) antigen NP-KLH before examining plasma cell (PC) and germinal center (GC) development.

While TD antigens generally do not favor plasma cell (PC) responses, we first asked whether RIP1 and caspase-8 deficient mice could expand their plasma cells (PC) following immunization with TD-antigen NP-KLH. Twelve days following immunization, we observed no changes in total PC frequencies in all mice tested. We observed no significant difference in *total* plasma cell frequency compared to unimmunized controls across the different strains 12 days after immunization with NP-KLH (**Supplementary Figure 4A**). Similarly, after a single immunization with the TD antigen (100μg NP-KLH), flow cytometry revealed that NP-KLH immunization induced a modest but not significant increase in *antigen specific* PCs compared to unimmunized controls in all strains of mice tested except for DKO and TKO mice (**Supplementary Figure 4B**).

**Figure 5:**
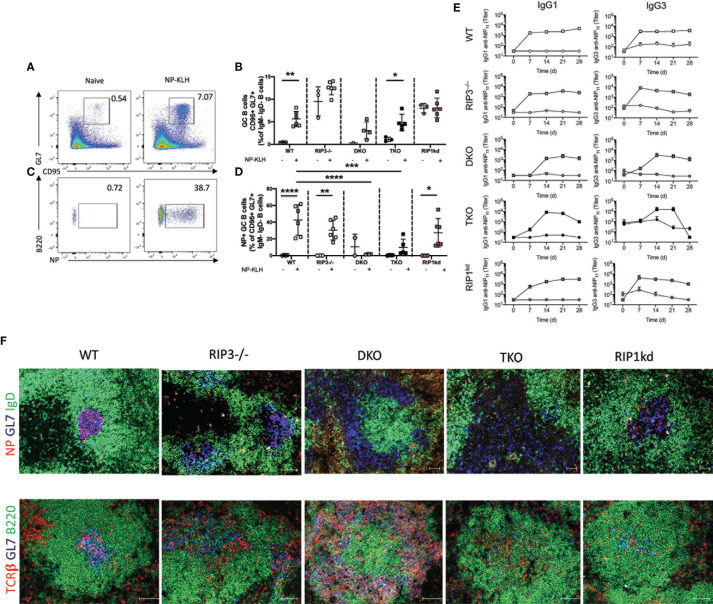
Caspase-8 is required for T-dependent B cell activation and maintenance of GC architecture. Representative flow cytometry plots demonstrate gating for frequency of splenic B220^+^CD19^+^ B cells which are GC B cells (GL7^+^ CD95^+^ IgM^-^IgD^-^) in naïve mice compared to mice immunized with 100μg NP-KLH and alum 12 days previously **(A)**. Total GC B cell frequencies quantified for naïve or immunized C57BL/6 WT, RIP3-/-, DKO, TKO and RIP1^kd^ mice **(B)**. Representative flow cytometry plots demonstrate gating for frequency of B220^+^CD19^+^ B cells which are NP-specific GC B cells (NP^+^GL7^+^ CD95^+^ IgM^-^IgD^-^) in naïve mice compared to mice immunized with 100μg NP-KLH and alum 12 days previously **(C)**. Antigen specific GC B cell frequencies quantified for naïve or immunized C57BL/6, RIP3-/-, DKO, TKO and RIP1^kd^ mice **(D)**. ELISA detected NP-specific IgG1 and IgG3 antibody titers in serum of C57BL/6 WT, RIP3-/-, DKO, TKO and RIP1^kd^ mice weekly for 28 days post immunization with 100μg of NP-KLH and alum **(E)**. Spleens from NP-KLH/alum immunized mice were labeled with immunofluorescent antibodies to identify the T cell zone (anti-TCRβ), B cell follicle (anti-B220), NP-specific B cells (NP-APC), and GCs (anti-GL7) **(F)**. Data is representative **(A, C, F)** or a pool **(B, D, E)** of at least two independent experiments, 3-4 mice per group. Each symbol indicates an individual mouse. *p ≤ 0.05, **p ≤ 0.01, ***p ≤ 0.001, ****p ≤ 0.0001 (one-way ANOVA) Scale bar = 100μm and represents 2-4 images per slide, 3-6 sections per mouse, and 4-6 mice per group **(F)**.

We next turned our attention to the splenic germinal center response. We used flow cytometry to determine the frequency of total and antigen-specific GC B cells 12 days after T-D antigen immunization (**Figures 5A–D**). Following immunization, the *total* GC B cell frequency was significantly higher in wild-type and TKO mice than in unimmunized controls (**Figure 5B**). Total GC B cell frequency also increased, but did not quite reach significance, following immunization in DKO mice (**Figure 5B**), while there was an unexplained high background of GC B cells in control unimmunized RIP3-/- and RIP1^kd^ mice which precluded detecting any increase in the total GC B cell response following immunization (**Figure 5B**). It is possible there was an ongoing response to self or foreign antigens in these mice from natural exposure which drove up the GC B cell frequency, but it did not affect the NP-specific GC B cell frequency. Examining the mice more precisely, we observed that TD antigen drove a significant expansion of antigen-specific GC B cells in wild-type, RIP3-/-, and RIP1^kd^ strains as expected, but not in DKO and TKO mice (**Figures 5C, D**). Next, we assessed the serological consequences of *Ripk1* and *Casp8* deficiencies after TD antigen immunization. To this end, we used ELISA to measure NP-specific IgG1 and IgG3 titers in serum over the course of 28 days post immunization with the TD antigen, NP-KLH. As expected, the wild-type mice showed early and robust NP-specific IgG1 and IgG3 responses which peaked at day 7 (**Figure 5E**). Peak NP-specific IgG1 antibody responses were delayed until day 14 in DKO mice while peak NP-specific IgG3 titers were delayed until day 21 in TKO mice (**Figure 5E**).

To consider the consequences of RIP1 and caspase-8-deficiency on GC structural organization, we used immunofluorescent imaging to identify splenic germinal centers. We found that RIP3-/-, DKO and TKO mice displayed disrupted GC architecture compared to wild-type mice (**Figure 5F**). Next, we stained follicular dendritic cell (FDC) networks with anti-CD35 to assess light zone (LZ, CD35^hi^) and dark zone (DZ; CD35^lo^) distribution. We found a concise separation of LZ and DZ in wild-type and RIP1^kd^ mice following immunization with NP-KLH. (**Supplementary Figure 4C**). Although, CD35^+^ FDCs were present in RIP3-/-, DKO, and TKO mice following NP-KLH immunization, they were mis-localized and no GL7^+^ or NP-specific cells were detected in proximity to FDCs (**Supplementary Figure 4C**). Thus, major alterations in the splenic architecture of DKO and TKO mice correlates with their inability to mount antigen-specific GC B cell responses (**Figure 5F**). Taken together, because the adaptive response phenotypes for DKO and TKO mice are nearly the same, these data show that caspase-8, but not RIP1, is required for timely B cell activation and germinal center response to TD antigens. Expansion of antigen-specific PCs also requires caspase-8 (**Supplementary Figure 4B**).

### *Ripk1*, *Casp8* Required For Antigen-Specific T-Independent Plasma Cell Expansion, but Not Antibody Response

MZ B cells mount critical early immune responses against pathogens, often mediated by recognition of T-independent (TI) antigens (45). Therefore, we assessed the impact of deficiencies in RIP kinases and caspase-8 on TI immune responses. We immunized RIP3-/-, DKO, TKO, and RIP1^kd^ mice with 30μg of the TI antigen NP-Ficoll (IP) and measured the innate B cell response. Twenty-eight days after the primary immunization we boosted the mice an injection of 30μg NP-Ficoll and then examined the spleens 17 days post-boost in comparison to the naïve controls.

**Figure 6:**
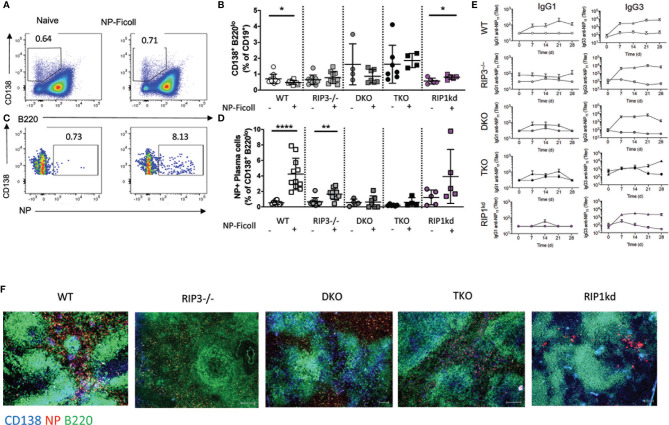
T-independent B cell responses require Casp8. Representative flow cytometry plots demonstrate gating for frequency of splenic CD19^+^ B cells which are plasma cells (PC; CD138^+^B220^-^IgM^-^IgD^-^) in naïve mice compared to mice immunized with 30μg of NP-Ficoll 28 days previously and 17 days after a boost **(A)**. Total PC B cell frequencies quantified for naïve or immunized C57BL/6 WT, RIP3-/-, DKO, TKO and RIP1^kd^ mice **(B)**. Flow cytometry plots demonstrating gating for frequency of splenic CD19+ B cells which are antigen specific (NP+) PCs **(C)**. Antigen-specific PC B cell frequencies quantified for naïve or immunized C57BL/6, RIP3-/-, DKO, TKO and RIP1^kd^ mice **(D)**. ELISA detects NP specific IgG1 and IgG3 antibody titers in serum of C57BL/6 WT, RIP3-/-, DKO, TKO and RIP1^kd^ mice weekly for 28 days after immunization with 30μg of NP-Ficoll **(E)** Spleens from NP-Ficoll immunized mice were labeled with immunofluorescent antibodies to identify B cell zone (anti-B220; green), PCs (anti-CD138; blue), and NP-specific B cells (NP-PE; red) in C57BL/6 WT, RI3-/-, DKO, TKO and RIP1^kd^ mice **(F)**. Data is representative **(A, C, F)** or a pool **(B, D, E)** of least two independent experiments, 3-6 mice per group. Each symbol identifies an individual mouse. *p ≤ 0.05, **p ≤ 0.01, ***p ≤ 0.0001 (One-way ANOVA) Scale bar = 200μm. Represents 3-4 images per slide, 3-4 sections per mouse, and 3 mice per group **(F)**.

We first evaluated the impact of RIP1 and caspase-8 deficiency on PC expansion. At steady-state, we found that both naïve DKO and TKO mice exhibited a significantly increased background frequency in *total* plasma cells compared to naïve wild-type mice (**Figures 6A, B**), consistent with the autoimmune syndrome first characterized by Kaiser et al. (18) and which we also confirmed (**Supplementary Figure 2**). Next, we measured frequencies of *antigen-specific* PCs. NP-Ficoll immunization induced significant or trending towards significant increases in frequency of antigen-specific PCs in wild-type, RIP3-/-, and RIP1^kd^ mice, as compared to naive controls (**Figures 6C, D**). Even though naïve DKO and TKO mice had increased background frequencies of total PCs compared to wild-type mice, neither DKO or TKO mice significantly expanded antigen-specific PCs in response to NP-Ficoll, when compared to their unimmunized controls. (**Figure 6D**).

We next used flow cytometry to determine the frequency of total and antigen-specific GC B cells in the spleen. As expected after immunization with NP-Ficoll, a largely plasma cell-stimulating, GC-independent TI antigen, the frequency of *total* GC B cells and *antigen-specific* GC B cells remained constant across different strains and did not significantly differ from unimmunized controls for all strains (**Supplementary Figures 5A, B**).

To determine how well antigen-specific PC expansion correlates with antigen-specific antibody production following immunization with the TI antigen, NP-Ficoll, we measured serum anti-NP IgG1 and anti-NP IgG3 titers over 28 days post immunization. Immunized wild-type mice showed an early antigen-specific IgG1 response to NP-Ficoll (**Figure 6E**). While DKO mice produced IgG3 antibody titers similar to wild-type mice in response to NP-Ficoll, TKO mice differed from wild-type controls in that they displayed a higher resting baseline of IgG3 than control mice and it did not increase with immunization (**Figure 6E**). This is consistent with the modest autoimmune syndrome in the DKO mice and the severe autoimmunity observed in the TKO mice (18). This also suggests broad innate and adaptive B cell defects in the TKO mice, but a more limited innate humoral defect in the DKO mice.

Next, we used immunofluorescence to determine whether RIP1 and caspase-8 are required for plasma follicle formation in the spleen 5 days after immunization with NP-Ficoll. Following immunization, extrafollicular NP-specific CD138^+^B220^+^ PC cells failed to accumulate in DKO mice when compared to all other genotypes. Surprisingly, TKO mice had modest but detectable antigen specific plasma follicles 5 days after NP-Ficoll immunization (**Figure 6F**). Thus, RIP1 and caspase-8 is required for total and antigen specific PC expansion. Proper organization of PC follicles require caspase-8. However, innate, TI B cell kinetics require RIP1.

Next, we used systemic (i.v.) lethal infection with *Streptococcus pneumoniae* as a means to evaluate the functionality of the MZ B cells in DKO and TKO mice. Because MZ B cells are essential for protection from systemic exposure to *S. pneumoniae* (46), mice with reduced MZB cell numbers (DKO) should have reduced survival compared with intact WT and mice with increased MZB cell numbers (TKO) should have increased survival compared to intact WT mice. To consider whether the increased numbers of MZ B cells in TKO mice were functional and conveyed a survival advantage over WT mice, we administered 6-10 x 10^5^ CFU/mL of *S. pneumoniae* i.v. to wild-type mice and the RIP kinase and caspase-8 deficient mouse strains. Compared to infected wild-type control mice, we found a significantly higher rate of survival by TKO mice following *S. pneumoniae* challenge (**Supplementary Figure 6A**). Surprisingly, DKO mice, which have a reduced frequency of splenic MZ B cells, did not succumb to infection at a higher rate than the wild-type mice (**Supplementary Figure 6A**). Taken together, these results suggest that RIP kinases and caspase-8 regulate the frequency and differentiation of MZ B cells, but the MZ B cells that do develop maintain normal effector function in the context of protection against lethal *S.pneumoniae* infection. Lethal *S.pneumoniae* challenge of DKO mice suggest that some, but not a full cohort, of MZ B cells is required for wild-type-level survival against systemic *S. pneumoniae* infection.

**Figure 7:**
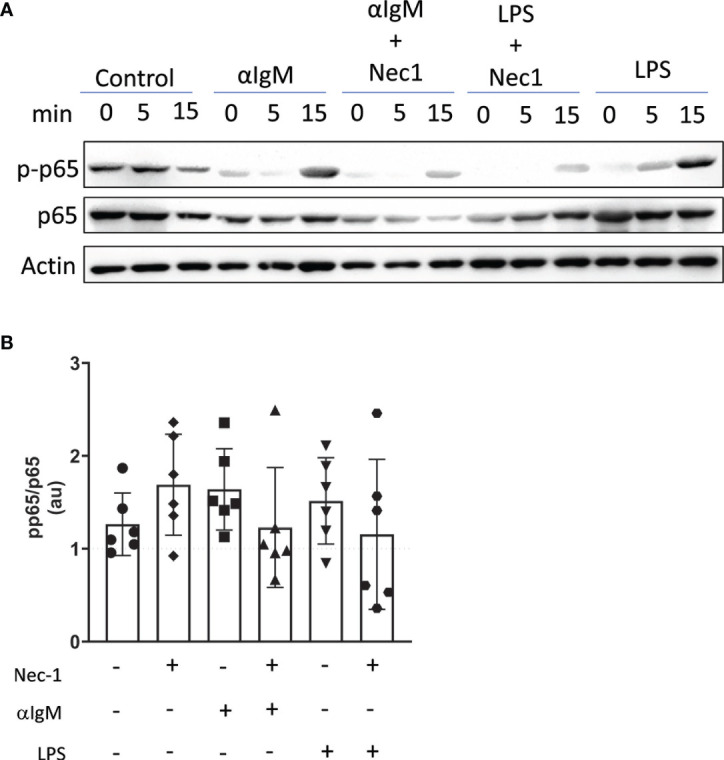
RIP1 inhibitor Necrostatin-1 reduces BcR mediated induction of p-p65. Immunoblot detected p-p65, p65, and β-actin protein in lysates from WT splenic B cells pretreated *in vitro* with or without 39.5 mM necrostatin-1 (Nec1) for 60 min and then stimulated with 15 mg/ml anti-Ig F(ab’)_2_ or 10 mg/ml LPS for 0, 5, 15 min **(A)**. Band intensity at 15min timepoint was quantified with Image J and is expressed as a ratio of pp65 to p65 arbitrary units (au) each normalized to actin **(B)**. Data is representative **(A)** or a pool **(B)** of six independent experiments, each with splenocytes from a single mouse. Each symbol identifies an individual mouse/experiment. (One way ANOVA, Friedman post-test).

Next, we used immunoblotting to determine if RIP1 conveys signals downstream of the BcR. To that end, we assessed levels of NFκB signaling molecules upon BcR engagement with/without RIP1 inhibition. Following negative isolation, total splenic B cells were engaged through their BcR by anti-IgM F(ab)’_2_ antibody or through TLR4 by LPS. RIP1 is well established to signal downstream of TLR4. Hence, we used LPS treated B cells as a positive control. LPS treated and BcR stimulated B cells have modestly higher levels of pp65, measured as ratio of total p65, when compared to unstimulated B cells (**Figures 7A, B**). However, pre-treatment with RIP kinase inhibitor Necrostatin-1 trends towards a reduction in accumulation of pp65 in B cells upon TLR4 and BcR engagement (**Figures 7A, B**). While it has been previously documented that RIP1 conveys signals downstream of TLR4 (9), pp65 reduction by Necrostatin-1 during BcR engagement also suggests that RIP1 may play a similar role downstream of the BcR to positively regulate NFκB signaling.

In summary, RIP1- and caspase-8 deficiencies skew innate B cell frequencies towards the MZ B cell and B1-b cells and disrupt splenic architecture. These differences are supported in part by increased BAFF production by macrophages. Interestingly, intrinsic RIP1 and caspase-8 maintain MZ B cell frequencies but do not dictate splenic architecture or autoantibody production, at least during early timepoints. Caspase-8 is also required to properly initiate and maintain T-dependent B cell immune responses and RIP3 is necessary for normal GC architecture. RIP1 and caspase-8 are essential for timely development of cognate antibodies in response to T-dependent B cell antigens whereas RIP1 seemingly is more critical to mount a T-independent immune response. Alterations in innate B cell frequencies, antibody responses, GC and PC phenotypes, and architectural changes in RIP deficient mice are consistent with RIP1 conveying signals downstream of the BcR.

## Discussion

Cell death is essential for the removal of autoreactive or defective cells during the course of B cell development, for optimal repertoire development and to maintain self-tolerance (47). As such, cell death provides an important checkpoint to prevent autoimmunity. RIP kinases together with caspases constitute some of the most important and influential inter-protein regulatory systems governing organ development and autoimmunity restriction. When proteins of the RIP kinase-caspase-regulatory system are only partially intact, as for DKO and TKO mice or patients afflicted with the CRIA syndrome, autoantibodies develop along with splenomegaly and lymphadenopathy (1, 18). In DKO and TKO mice, mature B cell populations develop, but with altered frequencies. This alteration in development suggests the RIPK pathway may partially mediate B cell selection. These DKO and TKO strains provide previously unavailable tools to dissect the precise contribution of these cell death pathway molecules to B cell development, differentiation, and function *in vivo*. *Ripk3* ^–/–^ mice largely resemble wild type mice immunologically, with an intact immune system and normal frequencies of B and T cell compartments (10). In wild-type mice, caspase-8 negatively regulates RIP1, so RIP1 will be unrestricted and possibly overactive in DKO mice lacking both RIP3 and caspase-8 (33). Conversely, TKO mice lack both RIP3 and caspase-8 in addition to lacking RIP1, so instead of enhanced RIP1 activity, TKO mice exhibit reduced/absent RIP1 activity. According to the affinity theory of B cell development, the developmental outcome for B cells depends upon the strength of BcR signals they receive at the transitional stage. During B cell development, BcR signals that are too strong or too weak will lead to elimination of transitional B cells by apoptosis. However, within the desired BcR affinity range, weaker signals give rise to MZ B cells, while strong signals give rise to FO B cells (48).

In the absence of caspase-8 in the DKO mice, RIP1 will be released from regulation and will be overactive. If RIP1 is conveying signals downstream of the BcR, then we would expect DKO mice with overactive RIP1 to have increased levels of p-p65 and NFκB signaling following BcR engagement. T2 precursor cells will perceive this over-reactivity as a strong BcR signal which will favor FO B cell accumulation. This is consistent with our observation that DKO mice have increased FO B cells, reduced MZ B cells.

Contrastingly, TKO mice will lack both caspase-8 and RIP1 which creates a RIP1-deficient environment. If RIP1 is conveying signals downstream of the BcR, then RIP1 deficiency would cause low levels of p-p65 and NFκB signaling, which the T2 precursors would interpret as a low BcR signal. B cells with weak BcR signals will develop into MZ B cells. This is consistent with our observation that TKO mice develop a higher frequency of MZ B cells than wild-type mice. In summary, DKO mice display a lower frequency of MZ B cells, indicative of a higher BcR signal, while TKO mice display a higher frequency of MZ B cells, consistent with a lower BcR signal. B cell phenotypes in the DKO and TKO mice support the hypothesis that RIP1 may contribute to B cell fate decisions as an intrinsic downstream mediator of BcR signaling.

Finally, B1 B cell development requires the strongest constitutive BcR signaling when compared to the BcR signal strength which drives MZ B cell and FOB differentiation (49, 50). DKO mice have higher levels of RIP1 signaling than wild-type animals, which correlates with enriched populations of MZ B cells, but they have a complete absence of B-1 B cells. On the other hand, TKO mice lack RIP1 signaling but have a relatively normal B-1 cell frequency, although it is skewed to the B1-b sub-population. Extrapolating from our results, this suggests that even over-active RIP1 may not sustain a BcR signal strong enough to drive B1 B cell development, and RIP1 deficiency does not dramatically reduce B-1 cells either, supporting a role for redundant signaling pathways driving B-1 B cell commitment.

One supporting candidate cytokine required to maintain innate B cells, including both B-1 and MZ B cells, is BAFF. Lymphocytes produce BAFF; however, only very potent stimuli are able to induce lymphocytic BAFF protein expression. Instead, our data suggests that myeloid cells are the most frequent BAFF producers in all strains tested. BAFF+ macrophages are reduced in DKO, which is consistent with the loss of B-1 cells in DKO mice. On the other hand, BAFF+ macrophages are increased in TKO, which is consistent with their increase in frequency in both B-1b and MZ B cells. BAFF is required for the transition step from T1 to T2 B cells, so despite alterations in T1 populations in some strains, the increases in serum BAFF observed in DKO mice, TKO mice, and B^DKO^, B^TKO^ chimeras may contribute to the maintenance of normal levels of T2 cells in all DKO and TKO mice, including the BM chimeras with B-cell restricted kinase deficiencies. The presence of autoantibodies in circulation and kidneys of DKO and TKO mice is an additional sign of autoimmunity and provides further confirmation of lymphocyte checkpoint disruption.

In addition to innate cell developmental defects, we found that RIPK and caspase-8 deficiency also disrupted splenic architecture. RIP3-/-, DKO, and TKO mice all had disrupted GC formation, as determined by immunofluorescent microscopy. Interestingly, mixed BMC which restricted RIPK pathway and caspase-8 deficiencies predominantly to B cells revealed that the RIP1/caspase-8 control of MZ B cell frequencies and splenic architecture was B cell-intrinsic, pointing to the role of RIP1 in cell fate decisions rather than B cell effector function. In addition, the fact that the MZ B cell imbalance is still observed in BMC with B-cell restricted deletion of RIP1, RIP3, or caspase-8, but in the presence of primarily wild-type macrophages, is consistent with macrophage BAFF supporting, rather than driving, the MZ B cell differentiation defects observed in TKOs.

Further testing revealed that both DKO and TKO mice had impairments in their adaptive humoral responses, but only the TKO mice showed reduced innate humoral responses. These findings indicate an increasingly sophisticated multi-layered regulation for the formation of adaptive immune responses. RIP1 is central to both the adaptive and innate arms of humoral immunity and plays a unique role in the innate response in B cells. The RIP3-/-, DKO, and TKO mice had highly disrupted germinal center structures which could in part underly the delay in antibody production in these mice. However, our *Streptococcus pneumoniae in vivo* infection studies showed that RIP1 deficiency doesn’t influence humoral immunity enough to reduce effector protection mediated by DKO or TKO MZ B cells.

Finally, our results establish a potential role for RIP1 to provide a signaling bridge between BcR engagement and intranuclear NFκB signaling. This relationship is consistent with *in vitro* studies documenting the expected increase in intracellular p-p65 following TLR4 ligation by LPS treatment and finding a similar increase following BcR engagement. As confirmation of specificity, increases in p-p65 following either TLR4 or BcR engagement were also reduced with the RIP1 inhibitor necrostatin-1. Further studies are needed to establish a more detailed map of the potential molecular relationship between RIP1 and BcR engagement during innate B cell differentiation in order to dissect the role of RIP1 in mediating B cell developmental fate decisions.

Intriguingly, RIP1^kd^ mice which lack the kinase function of RIP1, have a B cell phenotype which is virtually the same as wild-type mice. This suggests most or all of the phenotypic defects observed in TKO mice can be attributed to RIP kinase-domain-independent effects, most likely mediated by the scaffolding domain. This is the same domain mutated in the human autoimmune disease CRIA (1), suggesting mutations of RIP in humans may mediate their effects through a similar kinase-independent mechanism. The consequences of partial or complete genetic deficiency of the RIP-caspase-regulatory system are in many regards similar between humans and mice (1, 20). Lymphadenopathy, splenomegaly, and autoreactive antibody production are well established between-species homologous pathologies. However, the mechanisms which drive human immune system anomalies and autoimmunity during RIP-caspase-system deficiencies remain to be clarified.

Our results show that RIP kinases and caspase-8 jointly orchestrate B cell fate through a B cell-intrinsic mechanism. The expansion of innate B cells we discovered in TKO mice is supported by myeloid cells that produce the cytokine BAFF, present at super-physiological amounts in the RIP1 deficient mice. These results are directly relevant for crafting therapeutic approaches for CRIA patients with impaired RIP and caspase-8 pathways. However, the critical importance of these pathways to fundamental B cell biology likely influences other autoimmune conditions as well, including systemic lupus erythematosus, skin disorders, gut inflammation, etc. Thus, the present study raises the prospect of targeting B cell activation influenced by RIP1 and caspase-8 pathways to limit numerous different human autoinflammatory diseases.

## Data Availability Statement

The raw data supporting the conclusions of this article will be made available by the authors, without undue reservation.

## Ethics Statement

The animal study was reviewed and approved by UTHSCSA IACUC.

## Author Contributions

RP, TH, and EL contributed to conception and design of the study. RP, TH, JH, AMc, AMa, ED, AK, LD, and EL contributed to acquisition or interpretation of data. RP, TH, and EL drafted and revised the article.

## Funding

This work was supported by the Swedish Research Council (TH), the US National Institutes of Health (R01 AI132798 to EL), and the University of Texas Health Science Center at San Antonio (EL). Some data was collected in the Flow Cytometry Shared Resource Facility, which is supported by UT Health, NIH-NCI P30 CA054174-20 (CTRC at UT Health) and UL1 TR001120 (CTSA grant).

## Conflict of Interest

The authors declare that the research was conducted in the absence of any commercial or financial relationships that could be construed as a potential conflict of interest.

## Publisher’s Note

All claims expressed in this article are solely those of the authors and do not necessarily represent those of their affiliated organizations, or those of the publisher, the editors and the reviewers. Any product that may be evaluated in this article, or claim that may be made by its manufacturer, is not guaranteed or endorsed by the publisher.
