# Supplementary material for: Receptor Interacting Protein Kinase Pathways Regulate Innate B Cell Developmental Checkpoints But Not Effector Function in Mice
Source: Front Immunol. 2021 Dec 9;12:758407. doi: 10.3389/fimmu.2021.758407 (PMC8696004; doi:10.3389/fimmu.2021.758407)
Supplement: Supplementary file 1 [file Presentation_1.pdf]

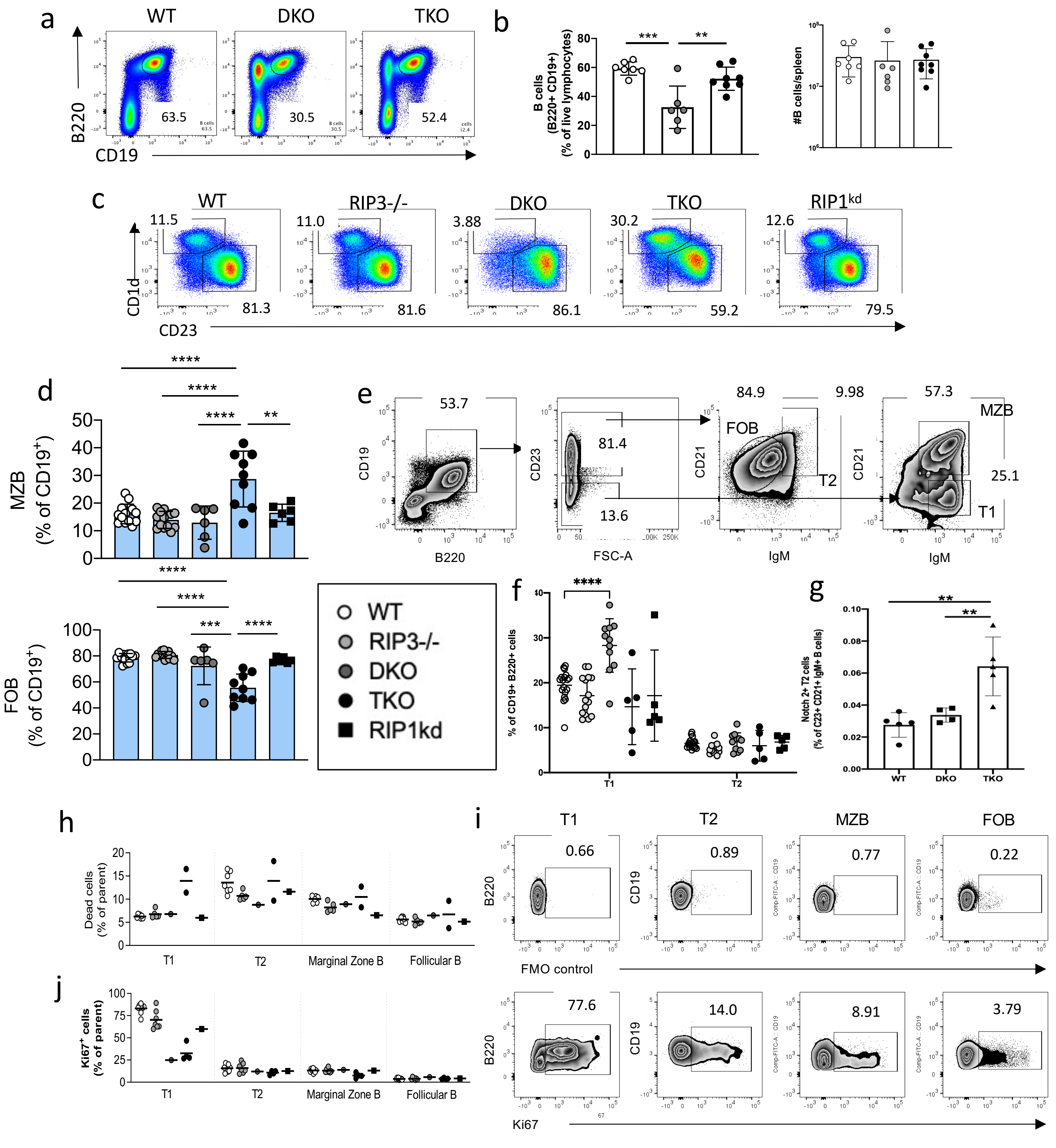

Supplementary Figure 1. RIP1/caspase-8 is required for innate B cell homeostasis

Flow cytometry of splenocytes from C57BL/6 WT, DKO, TKO mice reveals percent of B cells ($CD19^+$, $B220^+$) (a) Frequency of B cells in WT, DKO and TKO mice and number of B cells ($CD19^+B220^+$) per spleen in C57BL/6, DKO and TKO mice (b). Flow cytometry of splenocytes from C57BL/6 WT, RIP3-/-, DKO, TKO and RIP1kd mice reveals percent of B cells ($CD19^+$) which are MZB ($CD21^{lo}$, $CD1d^{hi}$) and FOB ($CD21^{hi}$, $CD1d^{lo}$) cells (c); quantified in (d). Representative Flow cytometry gating of C57BL/6 WT splenocytes identifies FO, MZ, T1 precursor ($CD23^-$, IgM^{hi}, $CD21^-$), and T2 precursor ($CD23^+$, IgM^{hi}, $CD21^+$) B cells (e). Quantification of T1 and T2 B cell precursor frequencies in C57BL/6 WT, RIP3-/-, DKO, TKO and RIP1kd mice (f). Frequency of Notch-2+ T2 precursor B cell in C57BL/6 WT, DKO and TKO mice (g). Frequency of T1 precursor, T2 precursor, MZ, and FO B cells which are dead in mouse strains as noted in key (h) Frequency of B cell subsets expressing Ki67 (bottom) as compared to FMO control (top)(i). Frequency of T1 precursor, T2 precursor, MZ, and FO B cells which are Ki67+ in mouse strains as noted in key(j). Data is representative (a,c,e,i) or a pool (b, d, f, g, h, j) of two independent experiments with 2-7 mice per group. Each symbol represents an individual mouse. *$p \leq 0.05$, **$p \leq 0.01$, ***$p \leq 0.001$, ****$p \leq 0.0001$ (One-way ANOVA and Unpaired two-tailed t-test).

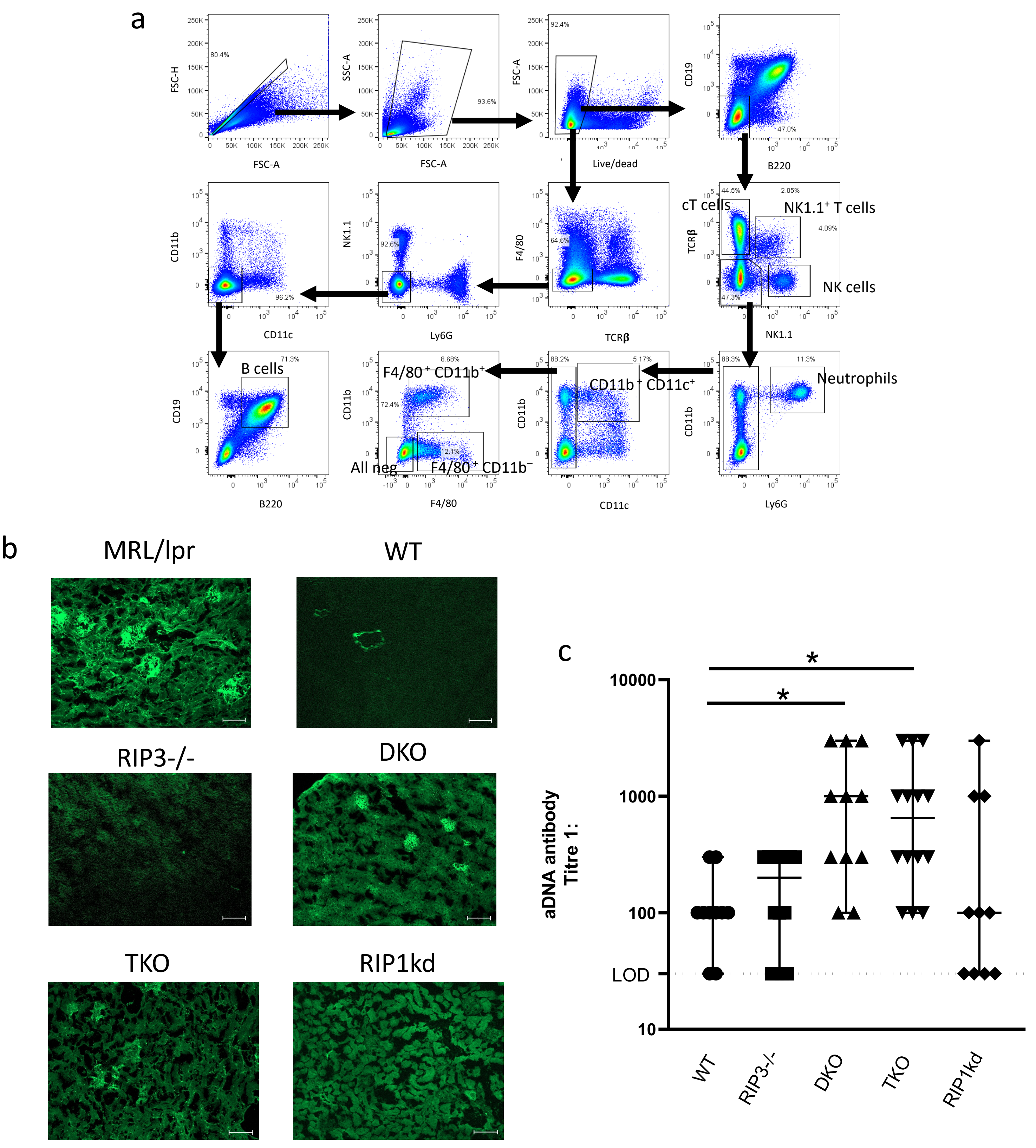

Supplementary Figure 2: DKO and TKO mice show signs autoimmunity

Flow cytometry plots showing gating strategy to identify myeloid populations (a). Immunofluorescent microscopy of 6µm kidney sections from 16-23 week old MRL/lpr, C57BL/6 WT, RIP3-/-, DKO, TKO, and RIP1kd mice labeled with mouse anti-IgG1 reveals autoantibody deposition (b). ELISA detects IgG2b anti-DNA antibodies in the serum of 16-23 week old C57BL/6, RIP3-/-, DKO, TKO and RIP1kd mice (c). Data is representative (a,b) or a pool (c) of at least 3 individual experiments with 4-6 mice per group. $^*p \leq 0.05$ (One way ANOVA and Unpaired student's t-test). Scale bar=200µm

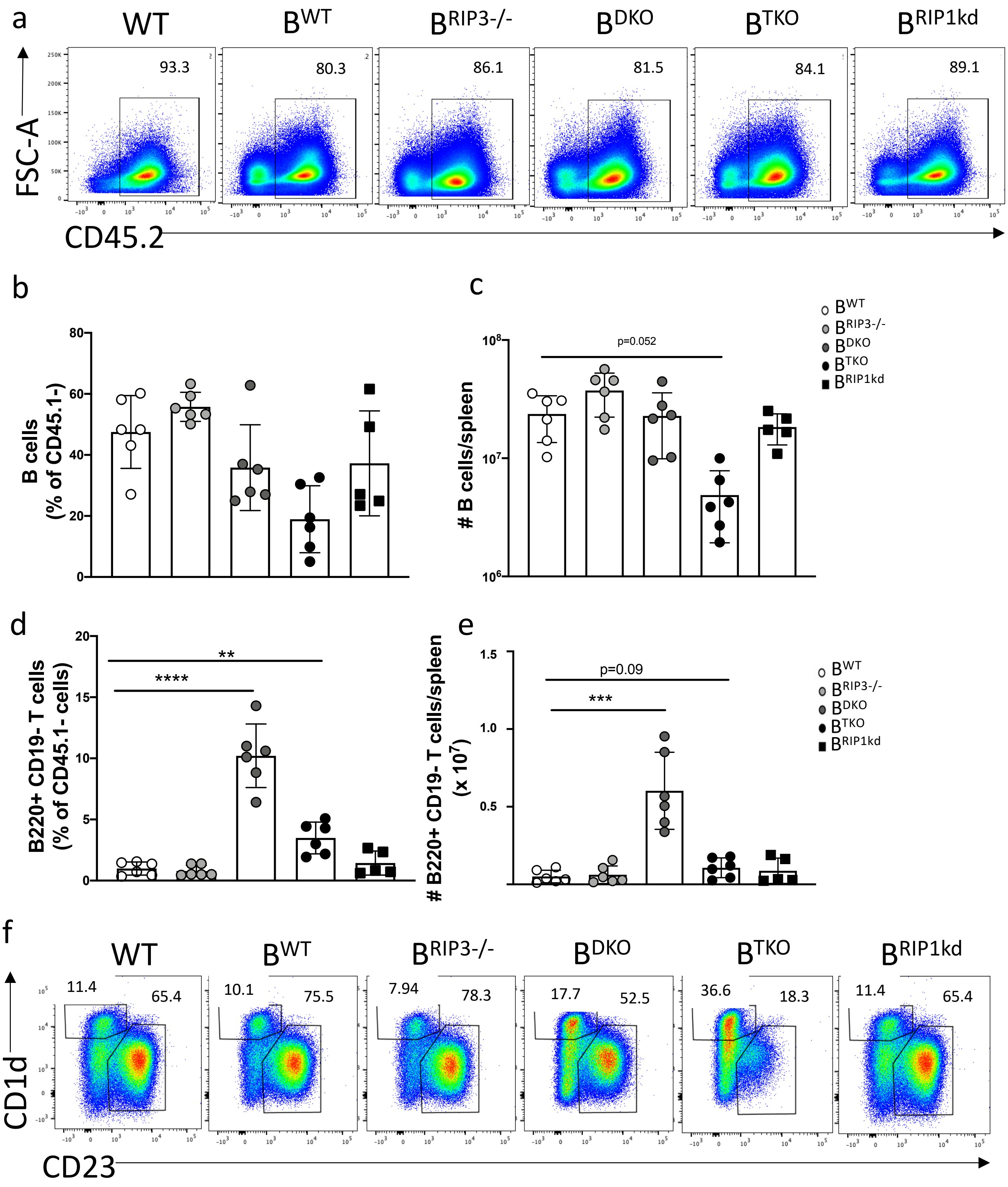

a
WT
BWT
BRIP3-/-
BDKO
BTKO
BRIP1kd
93.3
80.3
86.1
81.5
84.1
89.1
FSC-A
CD45.2
b
B cells (% of CD45.1-)
c
p=0.052
# B cells/spleen
BWT
BRIP3-/-
BDKO
BTKO
BRIP1kd
d
B220+ CD19- T cells (% of CD45.1- cells)
****
**
e
B220+ CD19- T cells/spleen (x 10^7)
p=0.09
***
f
11.4
65.4
10.1
75.5
7.94
78.3
17.7
52.5
36.6
18.3
11.4
65.4
CD1d
CD23

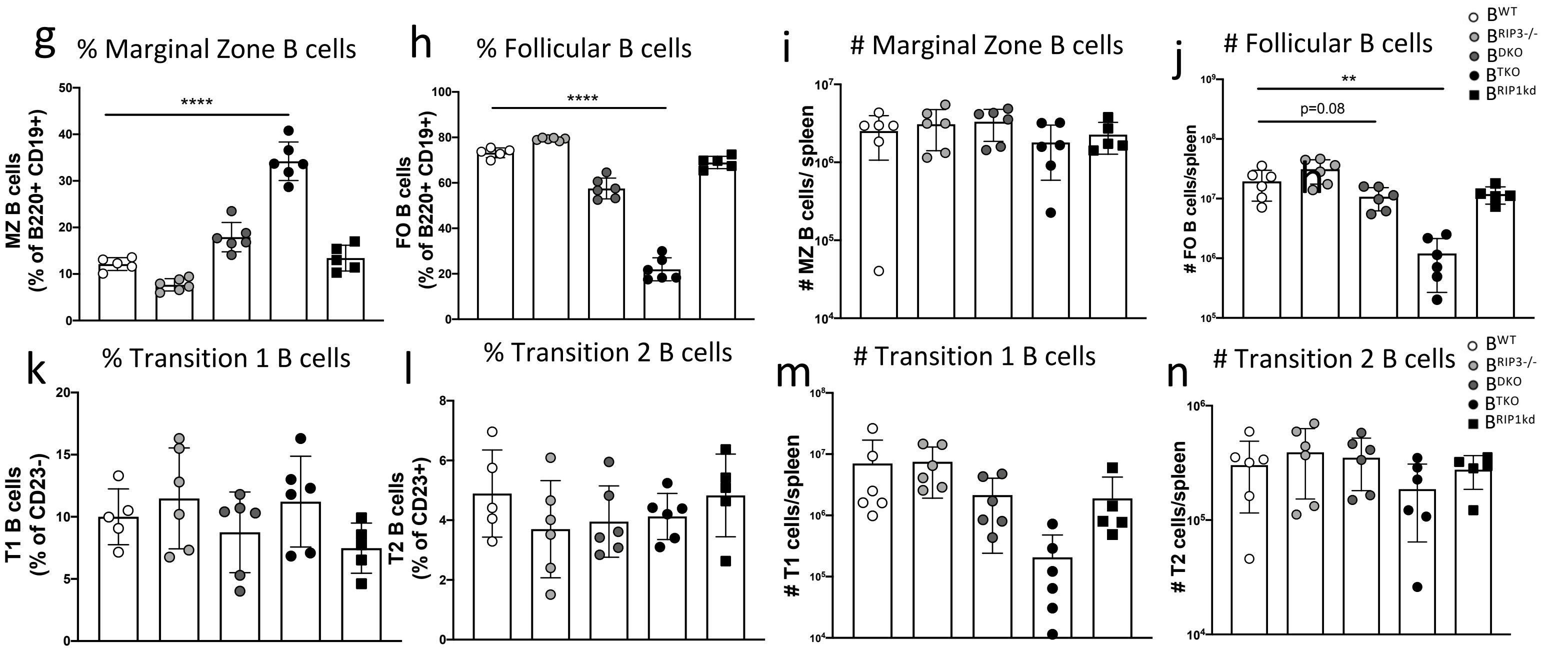

Supplementary Figure 3. Flow cytometric characterization of total spleen from C57BL/6 WT, B^{WT}, $B^{RIP3-/-}$, B^{DKO}, B^{TKO} and B^{RIP1kd} bone marrow chimeras 10 weeks post reconstitution. Percentages of cells in flow plot gates identify CD45.2+ donor bone marrow cells (a). Frequency (b) and number (c) of $B220^+$ $CD19^+$ cells in spleen of B^{WT}, $B^{RIP3-/-}$, B^{DKO}, B^{TKO} and B^{RIP1kd} mice. Quantification of $B220^+$ $CD19^-$ T cell frequencies (d) and numbers (e) per spleen in B^{WT}, $B^{RIP3-/-}$, B^{DKO}, B^{TKO} and B^{RIP1kd} mice. Representative flow cytometry gates capture frequencies of $CD19^+$ $B220^+$ $CD23^{lo}$ $CD1d^{hi}$ Marginal Zone B cells and $CD19^+$ $B220^+$ $CD23^{hi}$ $CD1d^{lo}$ FO B cells in spleens of WT, B^{WT}, $B^{RIP3-/-}$, B^{DKO}, B^{TKO} and B^{RIP1kd} mice (f). Quantification of frequencies (g, h) and numbers (i, j) of splenic MZB and FOB cells gated as in (f). Quantification of frequencies (k,l) and numbers (m,n) of $CD23^-$ IgM^{hi} $CD21^-$ Transitional type 1 B cell and $CD23^+$ IgM^{hi} $CD21^+$ Transitional type 2 B cell precursors. Data is representative (a, f) or a pool (b-e, g-n) of 3 independent experiments with 2-3 mice per group. Each symbol represents an individual mouse. *$p \leq 0.05$, ** $p \leq 0.01$, *** $p \leq 0.001$, **** $p \leq 0.0001$ (one-way ANOVA).

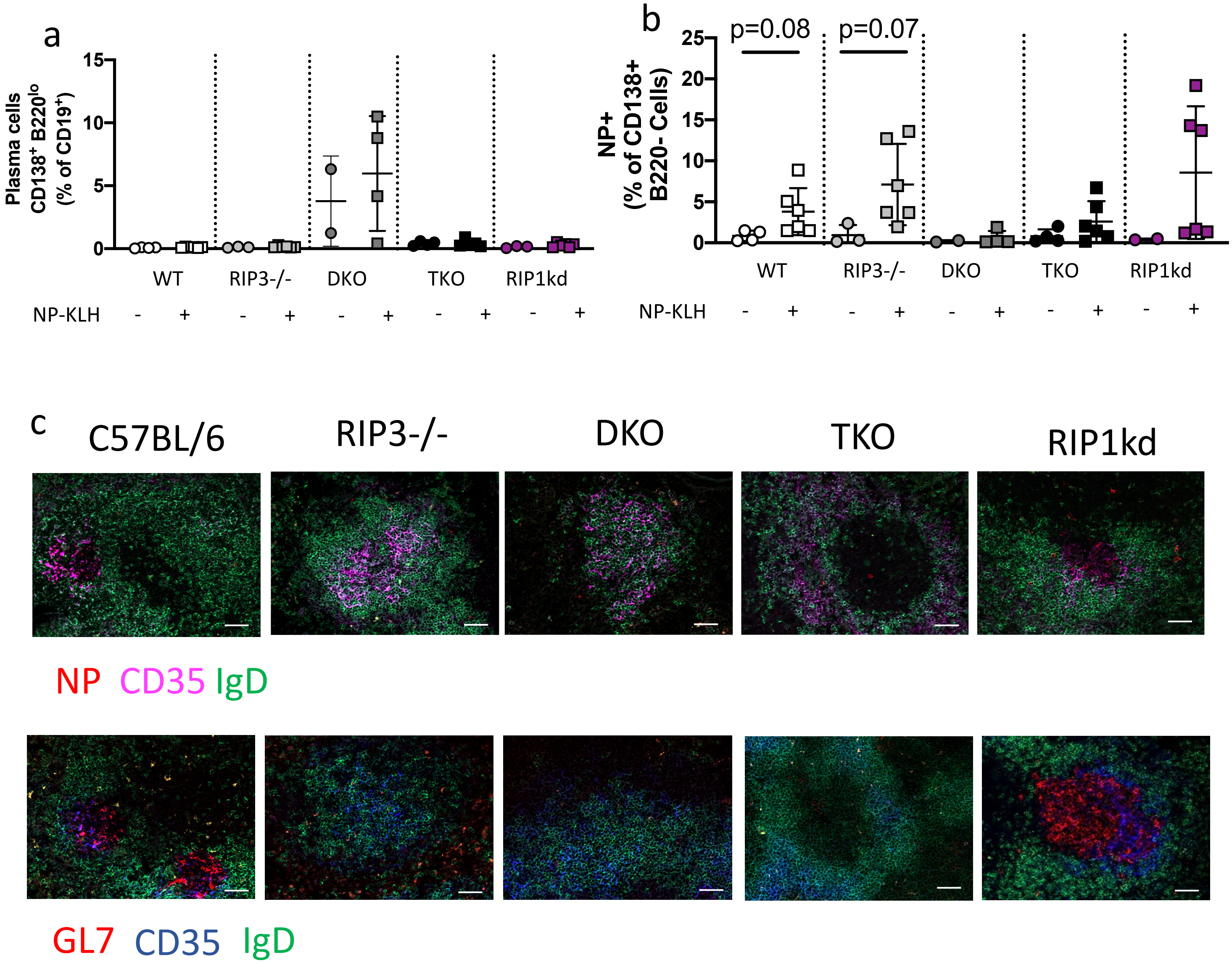

Supplementary Figure 4: DKO and TKO mice exhibit disrupted GC architecture following TD immunization. Flow cytometric quantification of total (a) and antigen specific (b) $CD138^{+}$ $B220^{-}$ plasma cells from 8-12 week old C57BL/6 WT, RIP3-/-, DKO, TKO and RIP1kd mice 12 days after immunization with 100μg NP-KLH and alum. Immunofluorescent images of spleens from C57BL/6 WT, RIP3-/-, DKO, TKO, and RIP1kd mice 12 days after immunization with NP-KLH/alum are labeled with NP-APC (red), anti-CD35 (pink), anti-IgD (green) (top) or GL7 (red), anti-CD35 (blue), and anti-IgD (green) (bottom) to identify antigen specific $NP^{+}IgD^{-}GL7^{+}$ germinal center B cells and $CD35^{+}$ follicular dendritic cells (c). Data is representative (c) or a pool (a,b) of three independent experiments with 2-3 mice per group. Each symbol indicates and individual mouse. *$p \leq 0.05$ (Two-way ANOVA). Scale bar=100μm

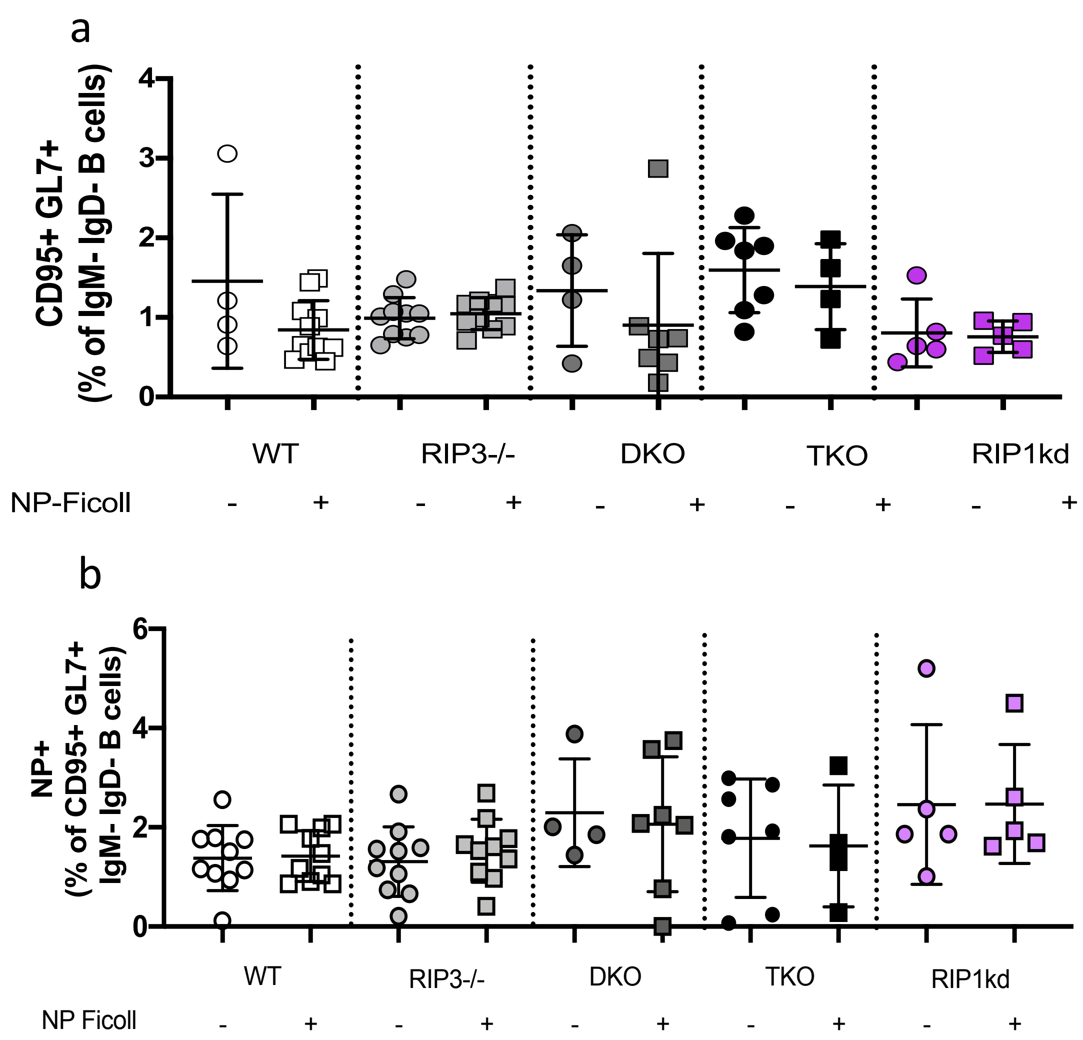

Supplementary Figure 5. Immunization with TI antigen does not expand GC B cells in any strains tested. Flow cytometric quantification of total (a) and antigen-specific (b) $CD95^{+}$ $GL7^{+}$ GC B cells from 8-12 week old C57BL/6 WT, RIP3-/-, DKO, TKO, and RIP1kd mice 17 days post boost with 30μg of NP-Ficoll. Data is pool of at least two experiments with 3-5 mice per group. Each symbol represents an individual mouse. *$p \leq 0.05$ (One-way ANOVA)

a

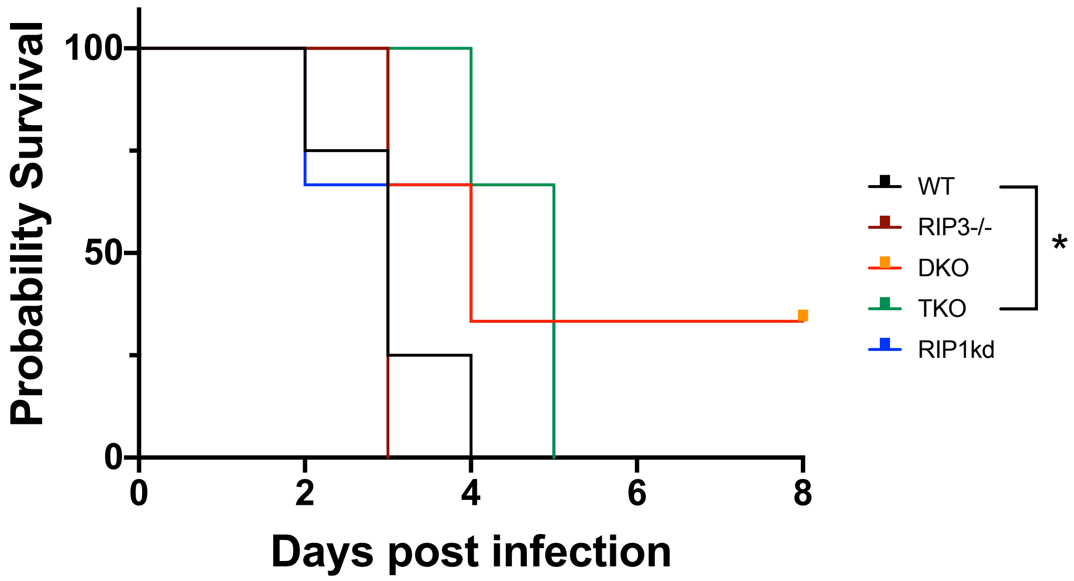

Supplementary figure 6. DKO mice survive systemic *Streptococcus pneumoniae* infection better than intact controls. Kaplan-Meier survival curve shows percent survival of C57BL/6 WT, RIP3-/-, DKO, TKO and RIP1kd mice injected IV with 6 x 10^5 CFU/mL URF 918 *S. pneumoniae* (a). Data is representative of two experiments with 3-4 mice per group. *$p \leq 0.05$ (Kaplan-Meier)

Supplemental table 1

Sl No	Type of mutation	Codon change	Amino acid change	Reported phenotype	References
1	Missense/nonsense	TAC-TAG	tyr212term	Immunodeficiency, IBD, growth failure and developmental delay	Uchiyama (2019) J Hum Genet 64, 995
2	Missense/nonsense	TAC-TAA	tyr426term	Immunodeficeincy and IBD	Li (2019) Proc Natl Acad Sci USA 116, 970
3	Missense/nonsense	TGT-TAT	cys601tyr	Immunodeficeincy and IBD	Li (2019) Proc Natl Acad Sci USA 116, 971
4	Missense/nonsense	ATT-ACT	ile615thr	Immunodeficeincy and IBD	Li (2019) Proc Natl Acad Sci USA 116, 972
5	Missense/nonsense	ACG-ATG	thr645met	Immunodeficeincy and IBD	Li (2019) Proc Natl Acad Sci USA 116, 973

Human Genome Mutation Database
